# Making the hydrogen evolution reaction in polymer electrolyte membrane electrolysers even faster

**DOI:** 10.1038/ncomms10990

**Published:** 2016-03-10

**Authors:** Jakub Tymoczko, Federico Calle-Vallejo, Wolfgang Schuhmann, Aliaksandr S. Bandarenka

**Affiliations:** 1 Center for Electrochemical Sciences—CES, Ruhr-Universität Bochum, Universitätsstrasse 150, D-44780 Bochum, Germany; 2 Analytische Chemie—Elektroanalytik & Sensorik, Ruhr-Universität Bochum, Universitätsstrasse 150, D-44780 Bochum, Germany; 3 Leiden Institute of Chemistry, Leiden University, PO-Box 9502, 2300 RA Leiden, The Netherlands; 4 Physik-Department ECS, Technische Universität München, James-Franck-Strasse 1, 85748 Garching, Germany; 5 Nanosystems Initiative Munich (NIM), Schellingstrassee 4, 80799 Munich, Germany

## Abstract

Although the hydrogen evolution reaction (HER) is one of the fastest electrocatalytic reactions, modern polymer electrolyte membrane (PEM) electrolysers require larger platinum loadings (∼0.5–1.0 mg cm^−2^) than those in PEM fuel cell anodes and cathodes altogether (∼0.5 mg cm^−2^). Thus, catalyst optimization would help in substantially reducing the costs for hydrogen production using this technology. Here we show that the activity of platinum(111) electrodes towards HER is significantly enhanced with just monolayer amounts of copper. Positioning copper atoms into the subsurface layer of platinum weakens the surface binding of adsorbed H-intermediates and provides a twofold activity increase, surpassing the highest specific HER activities reported for acidic media under similar conditions, to the best of our knowledge. These improvements are rationalized using a simple model based on structure-sensitive hydrogen adsorption at platinum and copper-modified platinum surfaces. This model also solves a long-lasting puzzle in electrocatalysis, namely why polycrystalline platinum electrodes are more active than platinum(111) for the HER.

## Abstract (web summary)

There is substantial research into minimizing platinum use in polymer electrolyte membrane electrolyzers. Here, the authors report that the hydrogen evolution activity of platinum(111) electrodes can be significantly enhanced by monolayer amounts of copper, which weaken the binding of hydrogen intermediates.

## Graphical abstract

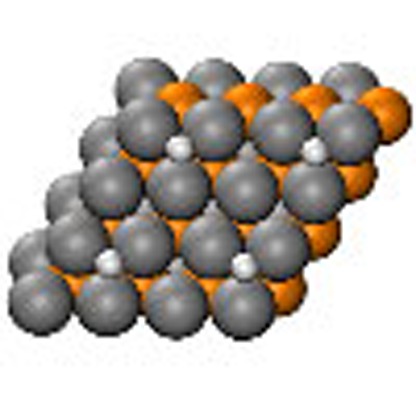

Heterogeneous redox reactions at electrified interfaces are of growing importance in contemporary science and technology^1^,^2^,^3^, as they determine the performance of several electrochemical devices for future sustainable provision, storage and redistribution of renewable energy^4^,^5^,^6^,^7^. In particular, the efficiency of electrolysers and fuel cells largely depends on their electrode/electrolyte interfaces and catalytic properties^8^,^9^,^10^,^11^.

In this context, hydrogen (H_2_) production from water is an important electrocatalytic process due to its dual impact: it is a good model catalytic system^12^,^13^,^14^,^15^ for the evaluation of new material design methodologies and it is significant for future energy provision and storage^16^,^17^,^18^,^19^. In spite of numerous achievements^18^, only ∼4% of H_2_ produced comes from water electrolysis^20^. The main impediments to a wider utilization of water electrolysis are the high energy losses in electrolysers due to the insufficient activity of state-of-the-art electrodes. Considering the global hydrogen production of ∼15 trillion moles per year (2011)^21^ and average prices (2016) in the United States and Europe of ∼0.1 Euro per kWh (refs 22, 23), the electricity costs to produce just 4% of H_2_ using polymer electrolyte membrane (PEM) electrolysers would exceed ∼6 billion Euros. Compared with these expenses for electricity, the material costs (noble-metal catalysts, supports and so on) are relatively small. For instance, decreasing the operating voltage of PEM electrolysers from presently ∼2.0 (ref. 18) by 0.1 V using improved hydrogen and oxygen evolution electrocatalysts could decrease the electricity expenses for electrolysis by ∼0.3 billion Euros. Assuming a current density in state-of-the-art PEM electrolysers of 1 A cm^−2^ (ref. 18) and catalyst loadings of 1 mg cm^−2^ for anodes and cathodes^18^, only the reduction in electricity expenses exceeds ∼10 times the whole price of platinum (with the amounts, which are equivalent to ∼0.5% of its annual production) or iridium (∼30% of its annual production, correspondingly) catalysts necessary to electrochemically produce the above-mentioned amount of hydrogen annually. In other words, a ∼20 mV decrease in the operating voltage of PEM electrolysers corresponds to the price of noble-metal catalysts needed to produce 4% of H_2_ electrochemically. Although the long-term goal is to replace scarce electrocatalysts with more abundant and similarly active analogues, fundamental and application-related issues require further optimization of state-of-the-art hydrogen evolution reaction (HER) and oxygen evolution reaction electrocatalysts^18^.

Here we show that incorporating (sub)monolayer amounts of copper (Cu) to platinum (Pt) enhances the catalytic activity ∼2 times at low overpotentials, surpassing the highest HER-specific activities reported under similar conditions. These results are rationalized in terms of a structure-sensitive analysis of hydrogen adsorption on Pt- and Cu-modified Pt surfaces that also explains why polycrystalline Pt is more active than Pt(111) towards the HER.

## Results

### General considerations

**Figure 1:**
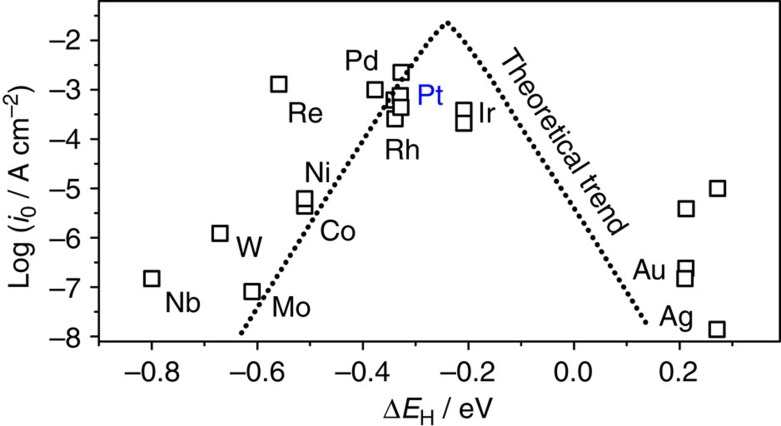
Trends in hydrogen evolution reaction activity. Experimental HER activity expressed as the exchange current density, log(*i*_0_), for different metal surfaces as a function of the calculated *H chemisorption energy, Δ*E*_H_. The result of a simple theoretical kinetic model is also shown as a dotted line. Original data are taken from ref. 25.

According to the current understanding, the HER (as well as hydrogen oxidation reaction, HOR) mechanisms involve adsorbed hydrogen (denoted *H) at the electrode surface. As stated by the Sabatier principle^24^, the optimal catalytic surface should bind reaction intermediates neither too weak nor too strong. This qualitative rule can be converted to a quantitative tool using calculated or measured adsorption energies for the relevant reaction intermediates at specific active sites on the surface. Figure 1 shows theoretical adsorption energies and experimental activity data for HER at pure metal surfaces^25^. As can be seen from Fig. 1, the trends in the measured HER can be fairly explained using the hydrogen binding energy as a ‘descriptor', Δ*E*_H_, estimated via density functional theory (DFT) calculations, as reported by Nørskov *et al*.^25^

**Figure 2:**
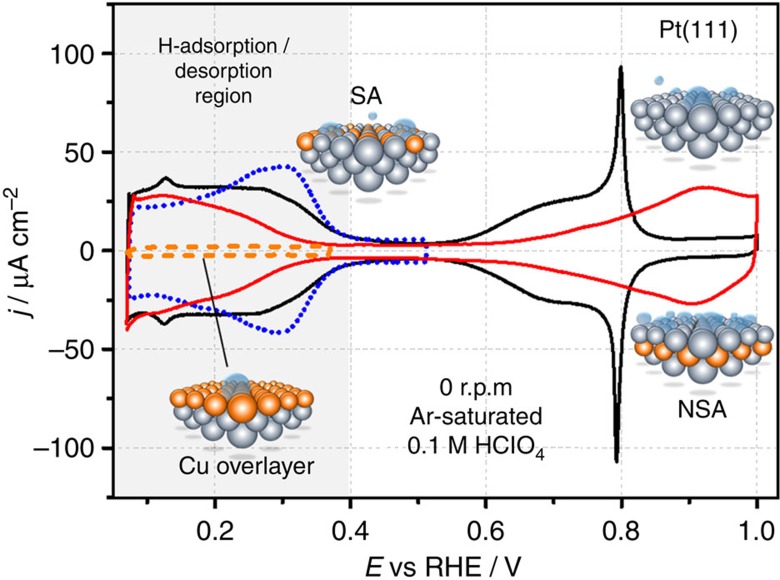
Cyclic voltammetry in Ar-saturated 0.1 M HClO_4_. The CVs of the Pt(111), Cu overlayer on Pt(111), Cu-Pt(111) SA and NSA (1 ML Cu initially deposited) demonstrate how the relative position of the Cu atomic layer governs the adsorption/desorption of hydrogen (grey area) at the electrode surface. d*E*/d*t*=50 mV s^−1^.

Although exact Δ*E*_H_ values depend on the surface coverage of hydrogen^12^, a straightforward outcome of this approach is that the optimum electrocatalytic sites for the HER should bind *H slightly weaker (∼0.09 eV) than Pd, Rh or Pt. In principle, the electronic properties of metal surfaces can be modulated by different means. One of the common ways to do this is to prepare bulk alloys, where the bulk crystal composition and structure influence the properties of the surfaces and, hence, their catalytic activity through strain and ligand effects^26^,^27^,^28^. An alternative way is to modify the properties of the topmost layer at the surface by selectively positioning atomic layers of solute metals directly at the surface to form either overlayers^29^, surface or subsurface alloys. An example of the latter approach is shown in Fig. 2.

### Electrochemical performance

Figure 2 shows cyclic voltammograms (CVs) taken in Ar-saturated 0.1 M HClO_4_ electrolytes for the unmodified Pt(111), the Cu overlayer on Pt(111), the Cu-Pt(111) near-surface alloys (NSAs) and surface alloys (SAs) within the regions of their electrochemical stability. In the CVs, the potential region between ∼0.4 and 0.07 V corresponds to hydrogen adsorption/desorption before the formation of H_2_ at more negative potentials. Notably, the position of the Cu atomic layer significantly changes the hydrogen binding energy. For example, the SA apparently binds *H stronger than Pt(111), as revealed by the corresponding CVs between ∼0.2 and ∼0.4 V (Fig. 2). In contrast, positioning the Cu atoms into the second layer (1 ML Cu initially deposited) weakens *H binding compared with unmodified Pt(111). In addition, the Cu pseudomorphic overlayer (POL) does not adsorb hydrogen species at these potentials (Fig. 2).

**Figure 3:**
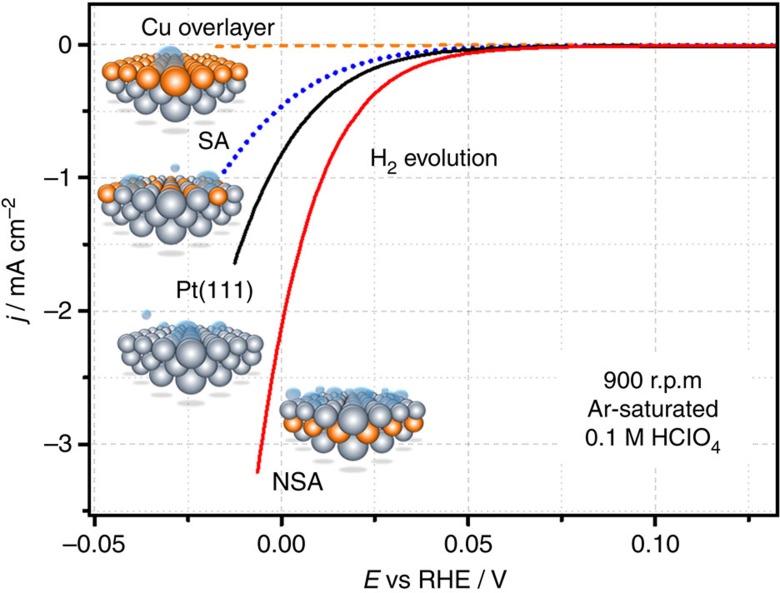
RDE voltammetry in Ar-saturated 0.1 M HClO_4_. The RDE voltammograms for Cu overlayer, Pt(111), SA and NSA (1 ML Cu initially deposited) electrodes show the correlation between the HER activity of the electrodes and the position of the Cu atomic layers relative to the topmost Pt layer. The negative currents start before 0.0 V RHE, because the electrolyte is saturated with the inert gas. d*E*/d*t*=10 mV s^−1^.

Experimentally observable changes in Δ*E*_H_ are mainly due to ligand effects, as Cu and Pt have dissimilar valence configurations (*s*^1^*d*^10^ versus *s*^1^*d*^9^) and the differences in the lattice constants are not negligible (3.61 versus 3.92 Å). These differences have a direct impact on the kinetics of reactions that involve adsorbed hydrogen species as reaction intermediates, in particular for the HER. For example, the SA, which binds hydrogen species stronger than Pt(111), would also probably be less active for both HER and HOR: this corresponds to the left part of the volcano plot in Fig. 1 and more negative Δ*E*_H_ values, relative to Pt. On the other hand, the Cu overlayer is ‘too noble' for the hydrogen species to be active towards HER. This corresponds to the right part of the volcano in Fig. 1, far from the optimum towards more positive Δ*E*_H_ values. In contrast, one can expect that the NSA would probably be more active than Pt(111): its surface binds hydrogen species slightly weaker than Pt, which corresponds to the direction towards the theoretical maximum in Fig. 1. In Fig. 3, we confirm all these expectations.

At low overpotentials, a Cu-Pt(111) POL does not show noticeable HER activities (Fig. 3). The SA is less active than Pt(111), as expected. Finally, the voltammogram for the NSA (1 ML Cu initially deposited) in Fig. 3 reveals a substantially higher hydrogen evolution activity as compared with that for Pt(111). It is noteworthy that the results presented in Fig. 3 correspond to measurements in Ar-saturated electrolytes, as these are the simplest tests to derive activity trends with minimal influence of complex experimental factors (those are especially important for overlayers, when the electrolyte is saturated with electroactive species such as H_2_ or CO)^30^. In the following, we focus on a more detailed electrochemical characterization of the active NSA electrodes.

**Figure 4:**
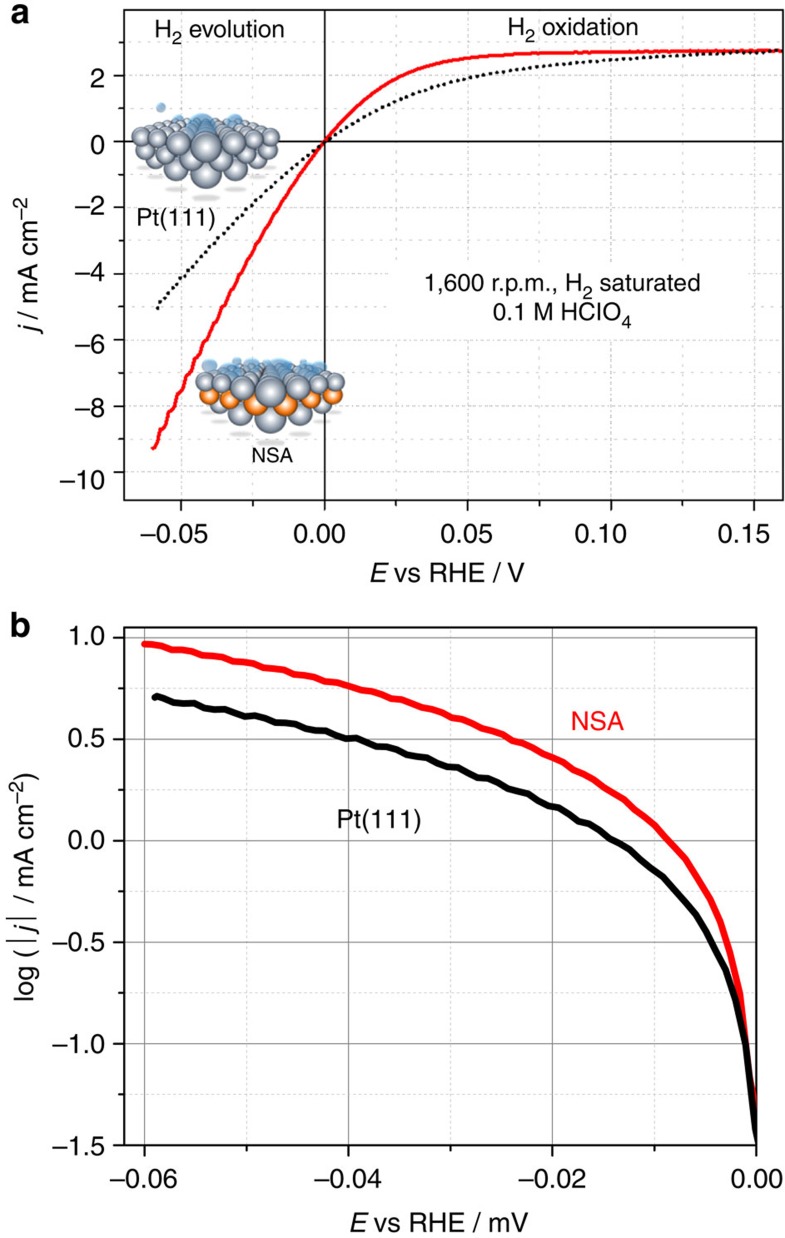
RDE voltammetry in H_2_-saturated HClO_4_. (**a**) RDE voltammograms of the Cu-Pt(111) NSA (1 ML Cu initially deposited) compared with the unmodified Pt(111) electrode. d*E*/d*t*=10 mV s^−1^. (**b**) Logarithmic plot of the currents related to hydrogen evolution for the NSA and Pt(111).

Figure 4 shows typical rotating disk electrodes (RDE) voltammograms recorded in H_2_-saturated 0.1 M HClO_4_ for Pt(111) and NSA electrodes. The NSA surface is more active than unmodified Pt for both HER and HOR. As those reactions involve the same intermediates, the same Δ*E*_H_ descriptor can be used to explain this fact. Although we do not use iR correction to avoid additional errors in this particular case (see ref. 30) and rather compare the model surfaces under the same conditions, it is still possible to approximately estimate the ‘apparent' exchange current density, *i*_0_, at very low overpotentials close to 0.0 V reversible hydrogen electrode (RHE)^15^. This value reflects the intrinsic activity of materials and can be used to compare different electrocatalysts reported by different research groups. The estimated *i*_0_ values are at least ∼1.5 mA cm^−2^ for Pt(111) and ∼3.0 mA cm^−2^ for the NSA. Notably, the apparent exchange current density for Pt(111) is higher than that reported in a very detailed investigation performed by Markovic *et al*.^15^ for low-index Pt(*hkl*) single-crystal surfaces measured in H_2_SO_4_ at the same pH value. We hypothesize that this is due to a difference in the exact experimental protocols, as discussed recently in detail in ref. 30. Nevertheless, this fact additionally prevents misinterpretation of the NSA activity results, as those are compared with already very active reference Pt surfaces.

If the so-called Tafel plot is used (Fig. 4b), the slopes of the curves for the NSA and Pt(111) samples at each electrode potential are rather similar, suggesting that there are no significant changes in the HER mechanism among these two surfaces.

Table 1 compares the activities for the HER/HOR at room temperature for active model surfaces, as summarized in refs 15, 18, 31 and measured in this work. In addition, as can be seen from Table 1, typical values for the apparent exchange current densities of Pt nanoparticles (averaged among values used by different groups), even at elevated temperatures and in real devices, are approximately three times lower than that for the NSA sample.

**Figure 5:**
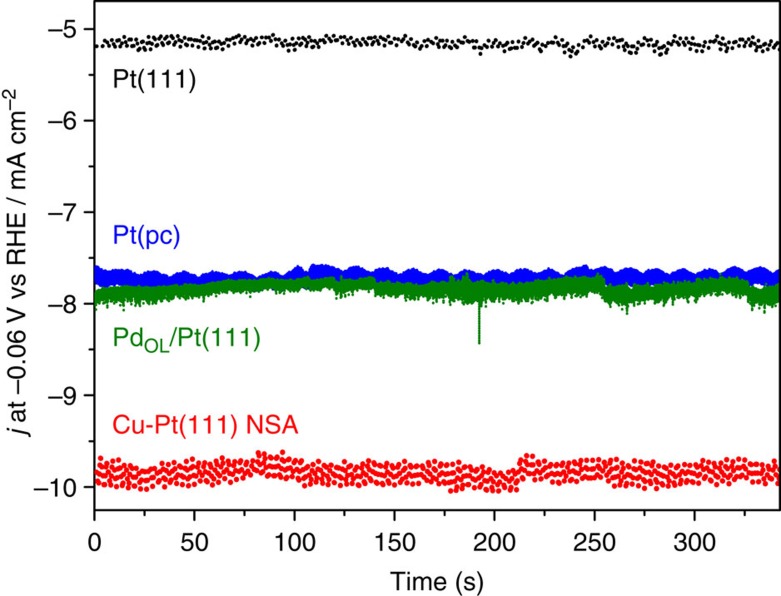
Hydrogen evolution activity as a function of time. The figure contains the current–time curves for hydrogen evolution reaction under potentiostatic conditions (*E*=−0.06 V) for the Cu-Pt(111) NSA, polycrystalline Pt, Pd_OL_-modified Pt and unmodified Pt(111) electrodes (RDE measurements at 1,600 r.p.m.).

To further evaluate the activity of the Cu-Pt(111) NSA with respect to the best known catalysts and additionally account for possible artefacts caused by the formation of the non-conducting H_2_ gas phase at the electrode surface during the cathodic/anodic scans, we compare chronoamperograms (current versus time curves taken at a certain potential) for the most active surfaces reported up to date in Fig. 5.

Figure 5 compares the activity of the Cu-Pt(111) NSA (1 ML Cu initially deposited) with polycrystalline Pt and Pd_OL_ deposited on Pt(111). First, the activity for all samples remains practically unchanged, as well as their basic CVs, indicating that the differences in activities are not due to artefacts caused by generation of the non-conducting gas phase. Notably, the activity of the Cu-Pt(111) NSA (1 ML Cu initially deposited) is reproducibly better than any other reported state-of-the-art electrocatalysts including polycrystalline Pt^32^, which has been suggested as one of the most active surface towards HER.

We performed additional benchmark measurements using polycrystalline Pt samples including iR correction. The activity results show that our polycrystalline samples possess exactly the same activity towards HER/HOR as reported by Sheng *et al*.^32^ in their detailed study of the activities of polycrystalline Pt^30^,^33^.

**Figure 6:**
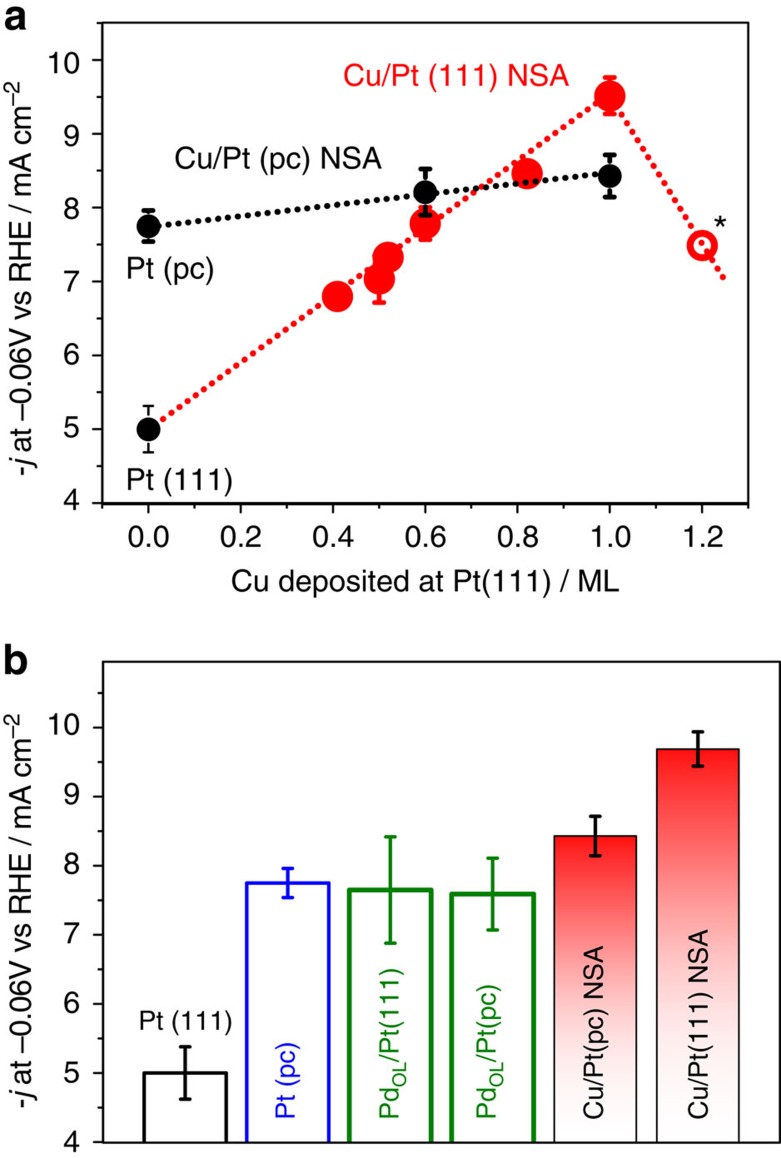
Effect of Cu and Pd addition on the hydrogen evolution reaction activity. (**a**) Effect of the Cu subsurface concentration on the HER activity measured at a potential of −0.06 V versus RHE for Pt(111) and polycrystalline Pt electrodes (Pt(pc), RDE measurements at 1,600 r.p.m., scan rate: 50 mV s^−1^), given with corresponding error bars (standard deviation) estimated using the results of at least five independent measurements. Open symbol with ‘*' represents the situation when more than 1 ML of copper was introduced intentionally. (**b**) Current densities measured at −0.06 V versus RHE for Pt(111), Pt(pc), Pd overlayer on Pt(111) (Pd_OL_/Pt(111), Pd overlayer on Pt(pc) (Pd_OL_/Pt(pc)), Cu/Pt(pc) NSA and Cu/Pt(111) NSA with corresponding error bars (standard deviation) estimated using the results of at least five independent measurements. The values are displayed without iR correction.

Furthermore, the influence of the subsurface concentration of Cu in Cu-Pt(111) and Cu-Pt(pc) NSAs has been tested by varying the amount of Cu initially deposited (Fig. 6a). Interestingly, the activity of the Cu-Pt(111) NSA sample with 1 ML Cu deposited initially remains the most active one. Attempts to introduce even more Cu into the subsurface region through a two-stage deposition/annealing procedure leads to a decrease in the activity (marked with ‘*' in Fig. 6a). All active samples evaluated in this work are compared in Fig. 6b.

Notably, the Cu-Pt(111) NSA samples demonstrated good stabilities towards H-induced segregation and anodic corrosion after 5,000 cycles between 0.05 and 1.0 V (versus RHE), as reported recently^34^. This additionally suggests that modification of just the subsurface region of HER electrocatalysts is a promising approach to enhance not only their catalytic activity but also their stability.

### Computational

**Figure 7:**
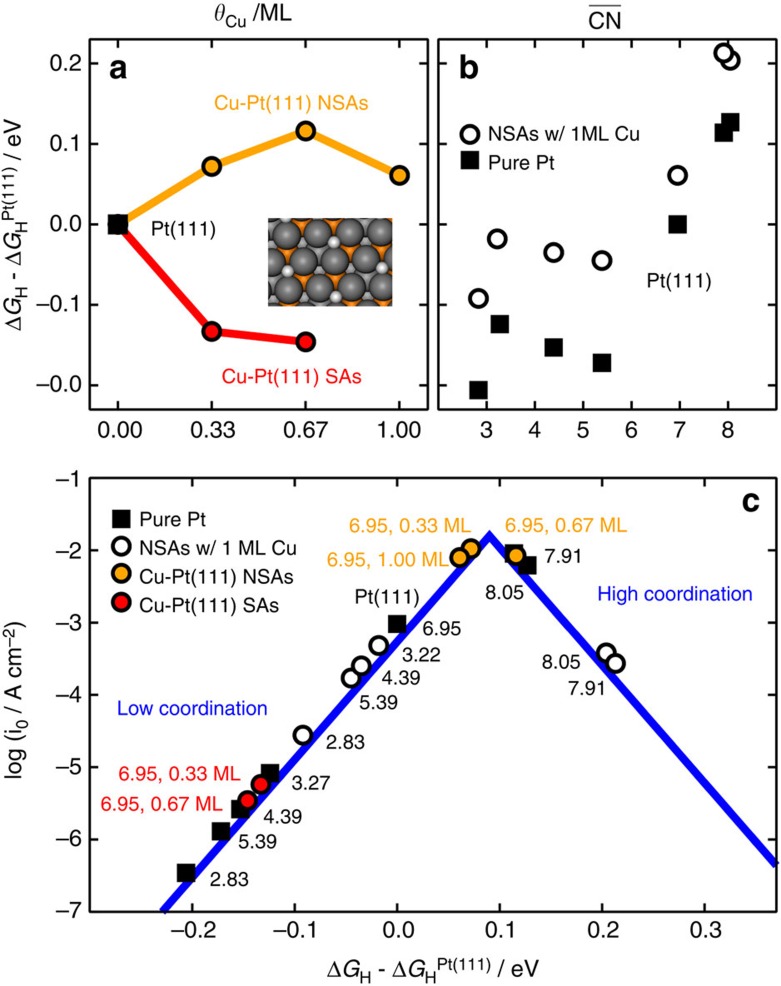
Trends in H_2_ adsorption and evolution. (**a**) *H adsorption energies with surface Cu (SAs, red) and subsurface Cu (NSAs, orange) on Pt(111). *H binds more strongly to SAs compared with Pt(111), whereas the opposite is observed for the NSAs. The inset shows *H at a NSA with 0.67 ML Cu (grey balls, Pt; red, Cu atoms; white, adsorbed *H). (**b**) Adsorption energies of *H on Pt and NSAs with 1ML Cu as a function of the generalized coordination numbers of the active sites. In all cases, *H adsorption energies are weaker on the NSAs than on the corresponding sites on Pt. Moreover, more coordinated sites than in the case of (111) terraces bind *H weaker, whereas the less coordinated ones bind it stronger. (**c**) Volcano plot showing the individual HER activity, coordination and composition of all studied sites. Sites at Cu-Pt(111) NSAs and overcoordinated Pt sites possess the highest activities, whereas SAs and undercoordinated sites are not active.

Finally, we rationalize our most important experimental findings based on the computational results as shown in Fig. 7. Figure 7a contains the trends in hydrogen adsorption for pure Pt(111) and CuPt(111) NSAs and SAs. Clearly, subsurface Cu at all concentrations has the same net effect of weakening the adsorption energies of atomic hydrogen, whereas surface Cu has the opposite effect in line with previous results^35^. DFT calculations confirm that hydrogen atoms are indeed bound more weakly at NSAs and more strongly at SAs.

To assess the structural sensitivity of the HER, we have tested the adsorption of *H at various sites apart from the hollow sites at (111) terraces usually considered in computational models^25^. Figure 7b shows the differences in adsorption energies of *H with respect to Pt(111) on numerous sites at Pt surfaces and NSAs with 1 ML Cu in the subsurface. The trends are described as a function of the generalized coordination numbers (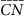) of the active sites^36^,^37^. In simple terms, generalized coordination numbers are a weighted average of the conventional coordination numbers. The weights are the coordination numbers of the nearest neighbours of the active sites. The various sites considered in this study and the way of estimating their generalized coordination numbers are provided in Supplementary Table 1.

Two noteworthy features of hydrogen atom adsorption on Pt and Cu-Pt NSAs are captured in Fig. 7b. First, sites with coordination lower than Pt(111) bind *H more strongly, whereas those with larger coordination bind more weakly. Second, *H adsorption on Cu-Pt NSAs is systematically weaker than on their counterparts at pure Pt, regardless of surface coordination.

Figure 7c contains the HER volcano-type activity plot built following the model by Nørskov *et al*.^25^ (see also Fig. 1 and Supplementary Methods). The plot reflects simultaneously the effect of geometric coordination and Cu content on the HER activity. First of all, highly coordinated defects on pure Pt are substantially more active than sites at Pt(111), which justifies the fact that polycrystalline Pt is more active than Pt(111) for the HER (see Supplementary Methods for further experimental evidence). Undercoordinated defects, however, are less active than (111) terraces. On the other hand, Cu-Pt(111) NSAs are highly active and both overcoordinated and undercoordinated defects decrease their activity, which explains why (111) NSAs are more active than the polycrystalline ones. Finally, SAs are not active in view of their strong *H adsorption energies. Therefore, structure- and composition-sensitive experimental trends for the HER in acidic media are well captured by the trends in *H adsorption energies. In turn, these energies are substantially influenced by the surface coordination of the active sites and the presence of Cu.

## Discussion

We have provided experimental and theoretical evidence to claim that selective positioning of Cu atomic layers modifies the adsorption properties of platinum electrodes for the electrochemical hydrogen evolution, accelerating one of the fastest electrocatalytic reactions known to date. Using predominantly the ligand effect, submonolayer amounts of Cu atoms in the second atomic layer induce a twofold increase in the electrocatalytic activity of Pt(111). This makes them the most active electrocatalysts ever reported for the HER in acidic media under comparable conditions, to the best of our knowledge. Further efforts to improve the performance of nanoparticle materials for the cathodes in PEM electrolysers may use this rationale based on the purposeful and delicate location of submonolayer amounts of foreign metals at surfaces.

## Methods

### Electrode preparation

Details relating to the electrode surface preparation and characterization are given in Supplementary Figs 1–13. The relative position of Cu atomic layers at the surface was controlled as described elsewhere^35^,^38^,^39^. Briefly, to form a copper POL or deposit submonolayer amounts of it, underpotential deposition was performed from a solution containing 2 mM Cu^2+^ in 0.1 M HClO_4_. The Cu-Pt(111) NSAs, where Cu atoms are preferentially located in the subsurface layer, were obtained by short annealing of the overlayer (∼2 min) at 400 °C in Ar/H_2_ atmosphere containing 5% of H_2_ in Ar (6.0, AirLiquide, Germany). Cu-Pt(111) SAs, where Cu atoms are located in the first atomic layer of the Pt host, were subsequently obtained by annealing the NSAs in Ar/CO atmosphere (0.1% CO in Ar, ∼2 min at 400 °C). The preparation procedures result in single-crystalline samples of Pt(111), Cu-Pt(111) NSAs and SAs, which were atomically smooth.

### Activity measurements

Electrolytes containing 0.1 M HClO_4_ (Merck Suprapur, Germany) were used for activity measurements. A mercury–mercury sulfate reference electrode was kept in a separate compartment and separated from the working electrolyte with an ionically conducting ceramic insert. A polycrystalline Pt wire was used as counter electrode. All potentials are referred to the RHE scale. A SP-300 potentiostat (Bio-Logic, France) was used to control the electrochemical measurements. Electrochemical experiments including the activity measurements were performed using a specifically designed electrochemical cell for the preparation and *in-situ* electrochemical characterization of single-crystal alloy electrodes, previously described in ref. 40. Measurements with RDE were performed using a Pine RDE 710 instrument (USA).

### DFT calculations

Full details of the DFT calculations, the assessment of adsorption energies, the model for estimating the current densities and the computation of generalized coordination numbers are provided in Supplementary Methods section with additional explanations illustrated in Supplementary Fig. 14 and Supplementary Table 1.

## Additional information

**How to cite this article:** Tymoczko, J. *et al*. Making the hydrogen evolution reaction in polymer electrolyte membrane electrolyzers even faster. *Nat. Commun.* 7:10990 doi: 10.1038/ncomms10990 (2016).

## Supplementary Material

Supplementary Information Supplementary Figures 1-14, Supplementary Table 1, Supplementary Methods and Supplementary References

## Figures and Tables

**Table 1:** Activities for the HER/HOR at room temperature.

Electrode	*i*_0,apparent_ (mA cm^−2^)*	Source
Pt(111)	∼0.45	ref. 15
Pt(100)	∼0.6	
Pt(110)	∼0.98	
Pd_OL_/PtRu(111)	∼2.0	refs 29,31
Pd_OL_/Pt(111)	∼2.0	
Pt(111)	∼1.5	This work
Cu-Pt(111) NSA (1 ML Cu initially deposited)	∼3.0	This work
Pt (nanoparticles)	∼1.0 (at 80 °C)	ref. 18

HER, hydrogen evolution reaction; HOR, hydrogen oxidation reaction.

^*^At pH 1, without iR correction, as reported in the literature.
