# Supplementary material for: Making the hydrogen evolution reaction in polymer electrolyte membrane electrolysers even faster
Source: Nat Commun. 2016 Mar 10;7:10990. doi: 10.1038/ncomms10990 (PMC4792955; doi:10.1038/ncomms10990)
Supplement: Supplementary Information — Supplementary Figures 1-14, Supplementary Table 1, Supplementary Methods and Supplementary References [file ncomms10990-s1.pdf]

<u>**Supplementary figures**</u>

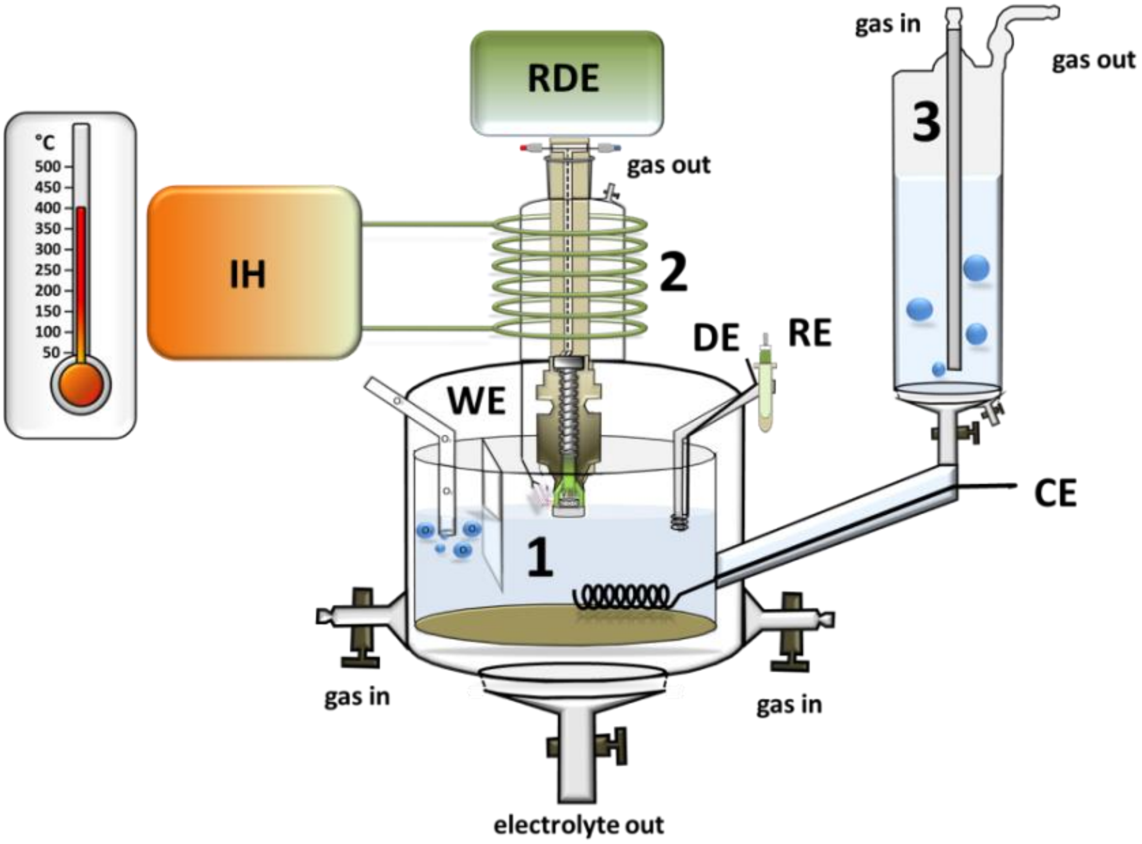


**Supplementary Figure 1.** Setup used for the preparation and characterization of Pt(111) electrodes. The cell consists of (1) the electrochemical compartment, (2) the high-temperature heat-treatment compartment, and (3) the compartment for electrolyte preconditioning. Abbreviations: IH – a high frequency inductive heater with an automatic time controller (20–80 kHz, 15KW - EQ-SP-15A, MTI), RDE – a rotating disk electrode motor, WE, RE, CE, DE are working, reference, counter and “dummy” electrodes, respectively. Reproduced by permission of the PCCP Owner Societies.

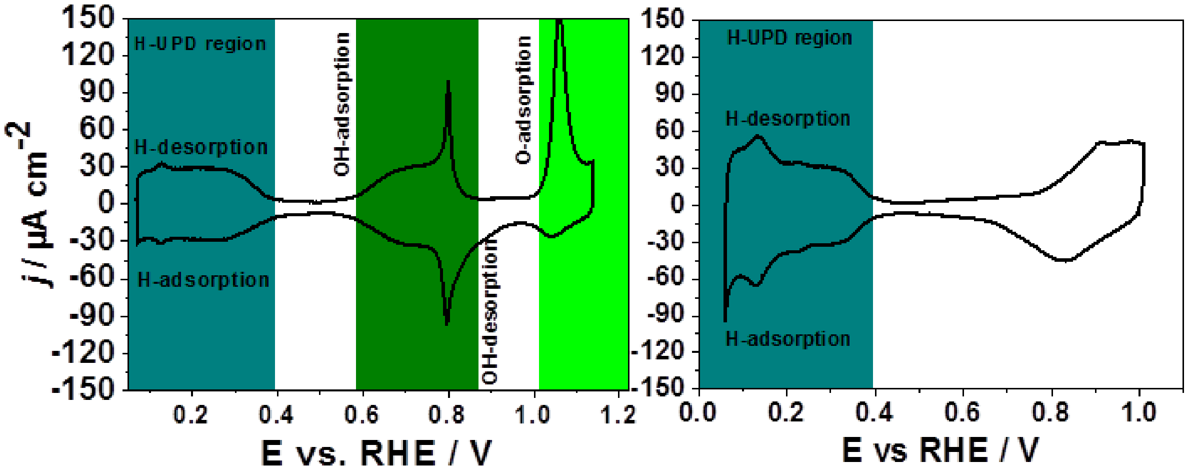


**Supplementary Figure 2.** Examples of cyclic voltammograms of freshly prepared Pt(111) (left) and Pt(pc) (right) electrodes. Scan rate d*E*/d*t* = 50 mV $s^{-1}$, Ar-saturated 0.1 M $HClO_4$.

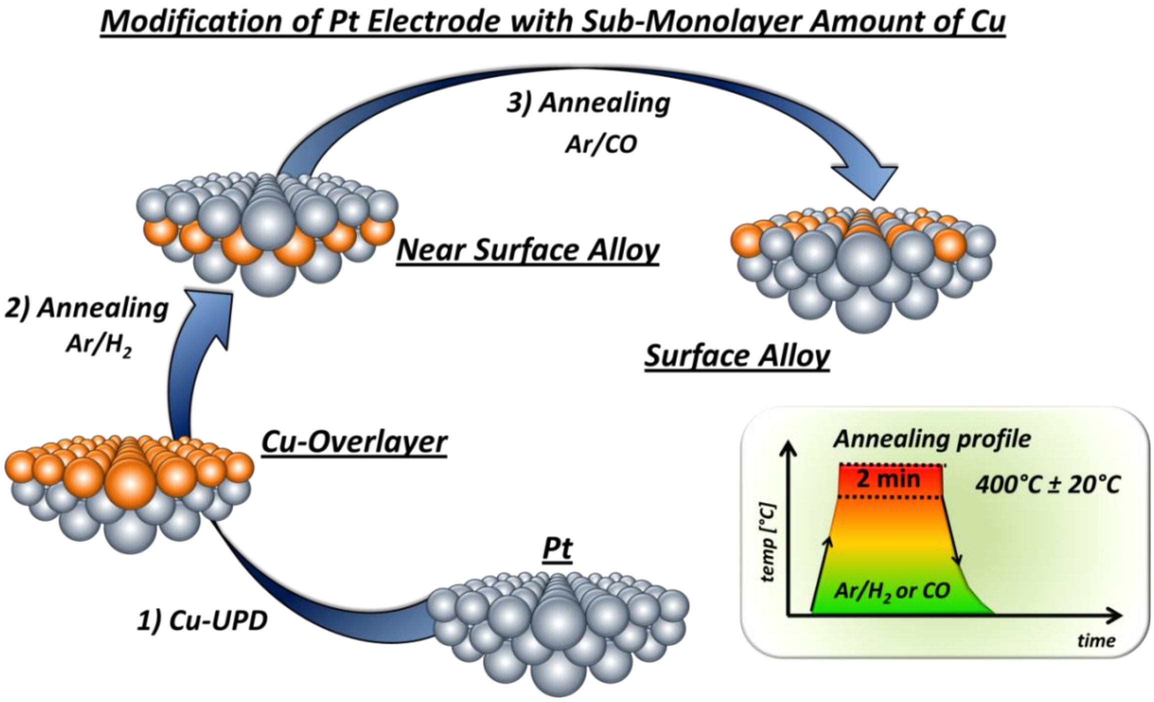


**Supplementary Figure 3.** Schematic representation of the procedure used to modify the Pt electrodes with submonolayer amounts of Cu. 1) Underpotential deposition of Cu at 0.32 V vs RHE for 3 min in Ar-saturated 0.4 mM $Cu^{2+}$ in 0.1 M $HClO_4$, 2) annealing at 400°C for 2 min in Ar/$H_2$ (10% $H_2$) and 3) in Ar/CO (0.1% CO) atmosphere to obtain a near-surface or a surface alloy, respectively.

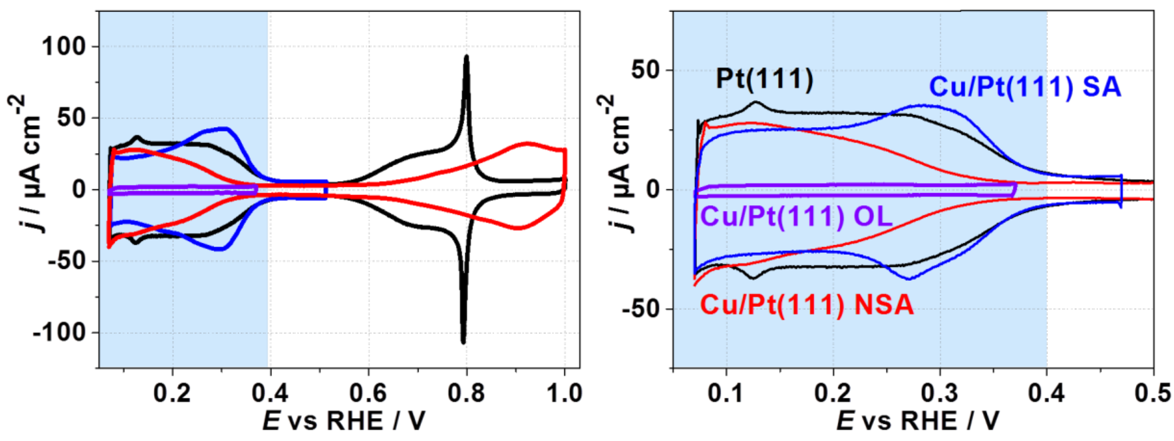


**Supplementary Figure 4.** Cyclic voltammograms of freshly prepared Pt(111), Cu overlayer on Pt(111) (OL), Cu-Pt(111) surface alloy (SA), and near-surface alloy. For NSA, 1 ML Cu was initially deposited. The voltammograms were obtained in Ar-saturated 0.1 M $HClO_4$, scan rate dE/dt = 50 mV $s^{-1}$.

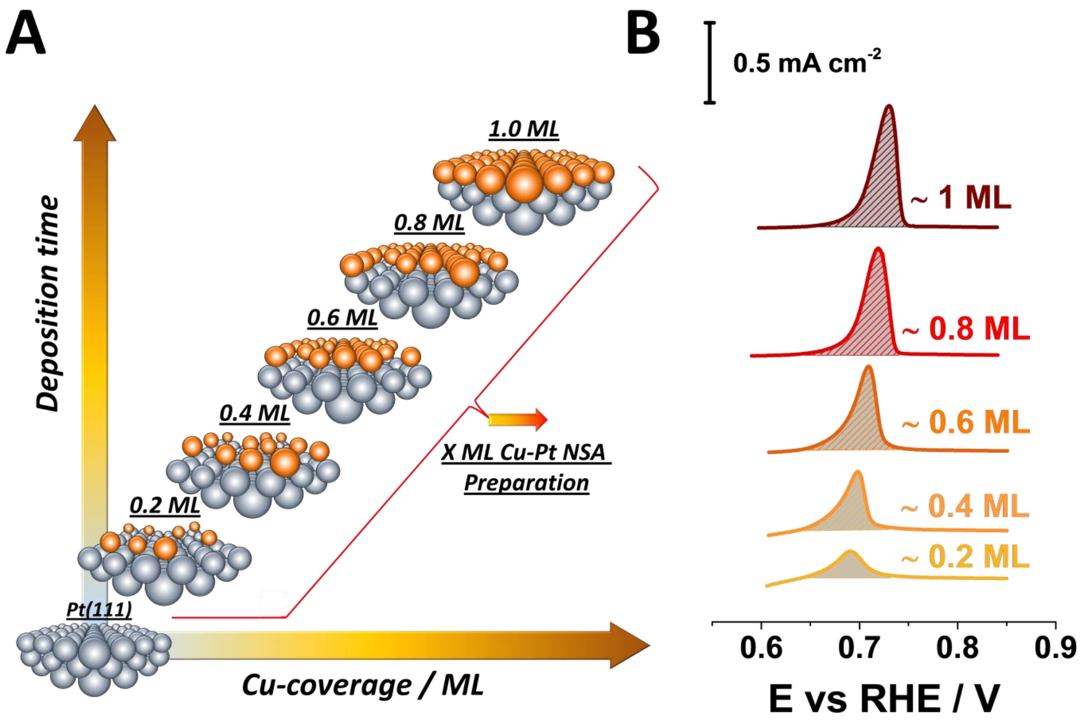


**Supplementary Figure 5.** Representation of the strategy used for preparation of Cu-Pt(111) near-surface alloys with different Cu concentrations in the second atomic layer. A) Schematics and B) Cu-oxidation peaks from Pt(111) in Ar-saturated 0.1 M $HClO_4$; scan rate d*E*/d*t* = 50 mV $s^{-1}$.

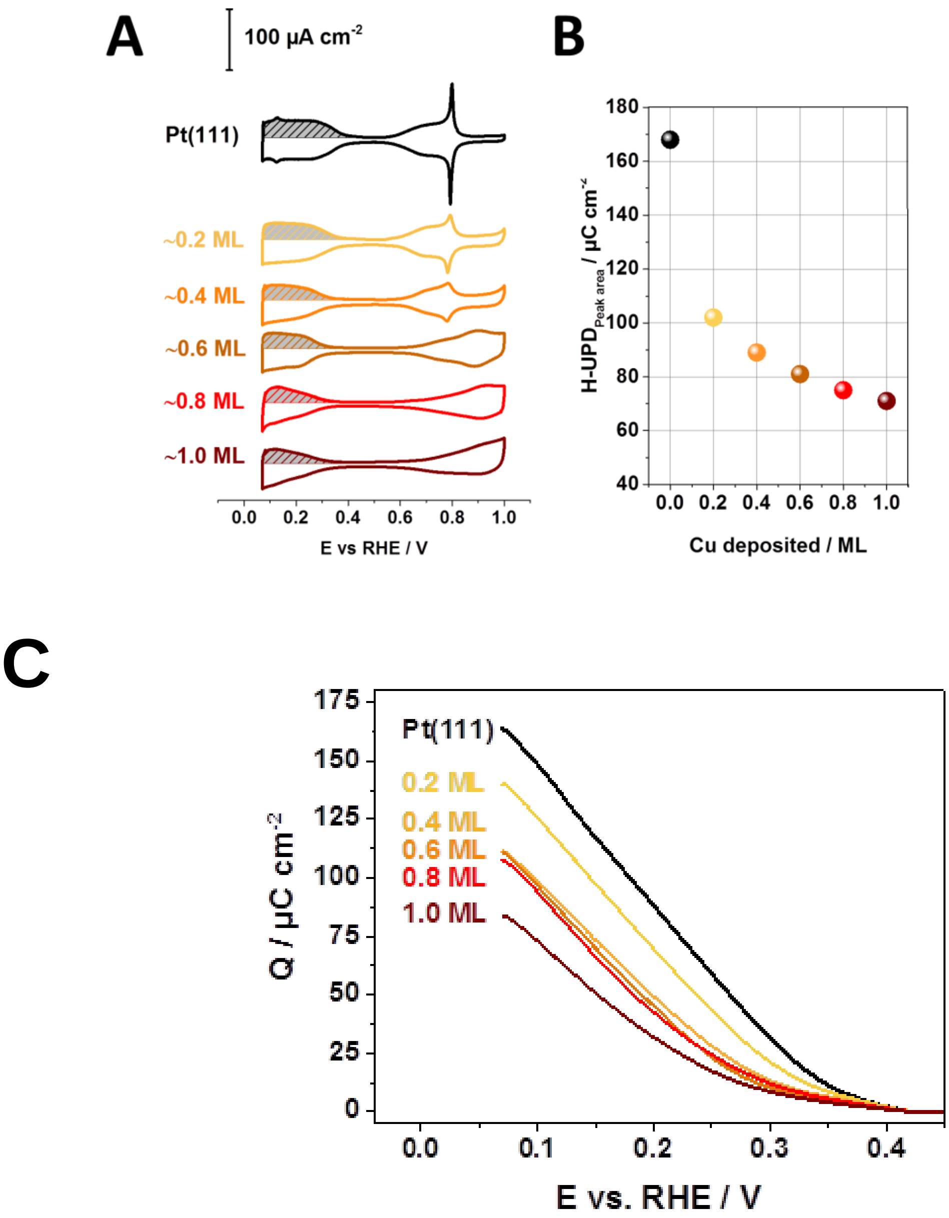


**Supplementary Figure 6**. Characterization of Pt(111) and Cu-Pt(111) NSA electrodes. A) Voltammetric characterization of a Pt(111) single-crystal electrode with different amounts of Cu in Ar-saturated 0.1M $HClO_4$, d*E*/dt = 50 mV $s^{-1}$. B) Correlation between the integrated H-UPD peak area (μC $cm^{-2}$) and the Cu amount (ML) deposited on Pt(111) electrode before preparation of Cu-Pt(111) NSA. C) integrated parts of the voltammograms (corrected for the double-layer charging) in the H-UPD region.

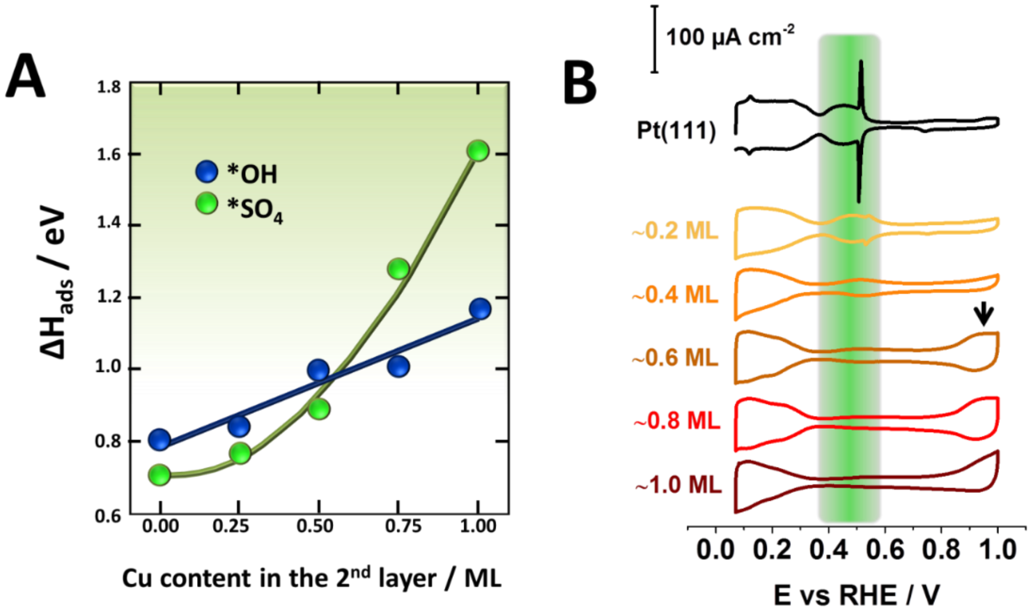


**Supplementary Figure 7**. Trends in adsorption energies for different electrode surfaces. A) Adsorption enthalpies for $*SO_4$ (green) and *OH (blue) on Pt(111) and Cu-Pt(111) NSA surfaces as a function of the Cu content in the subsurface layer. B) Cyclic voltammograms characterizing Pt(111) with different Cu content in the subsurface layer in Ar-saturated 0.05 M $H_2SO_4$, scan rate dE/dt = 50 mV $s^{-1}$. Arrow indicates the weakening effect of subsurface Cu on the adsorption energetics of $*SO_4$ favoring the adsorption of *OH.

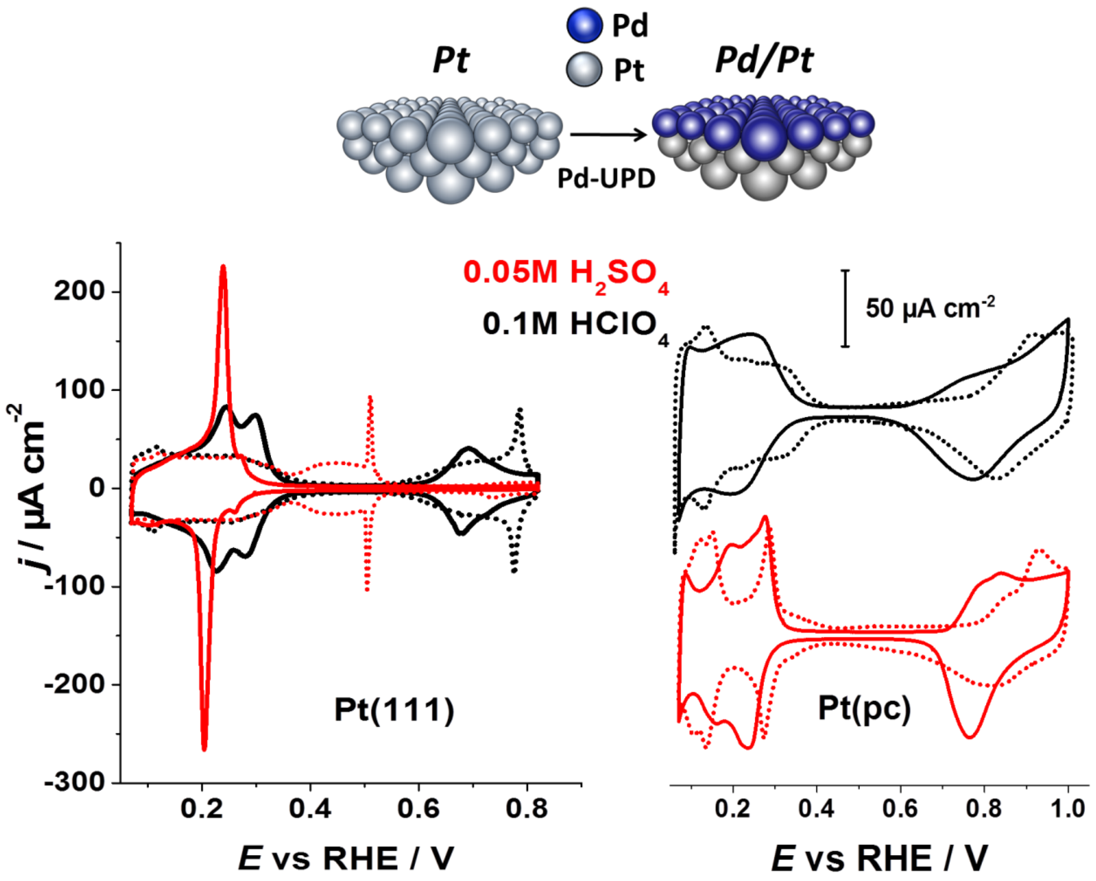


**Supplementary Figure 8**. Cyclic voltammograms for Pd overlayers. The voltammograms are shown for Pd overlayers on Pt(111) (left) and polycrystalline, Pt(pc), (right) in Ar-saturated 0.05 M $H_2SO_4$ (red) and 0.1 M $HClO_4$, scan rate d$E$/dt = 50 mV $s^{-1}$.

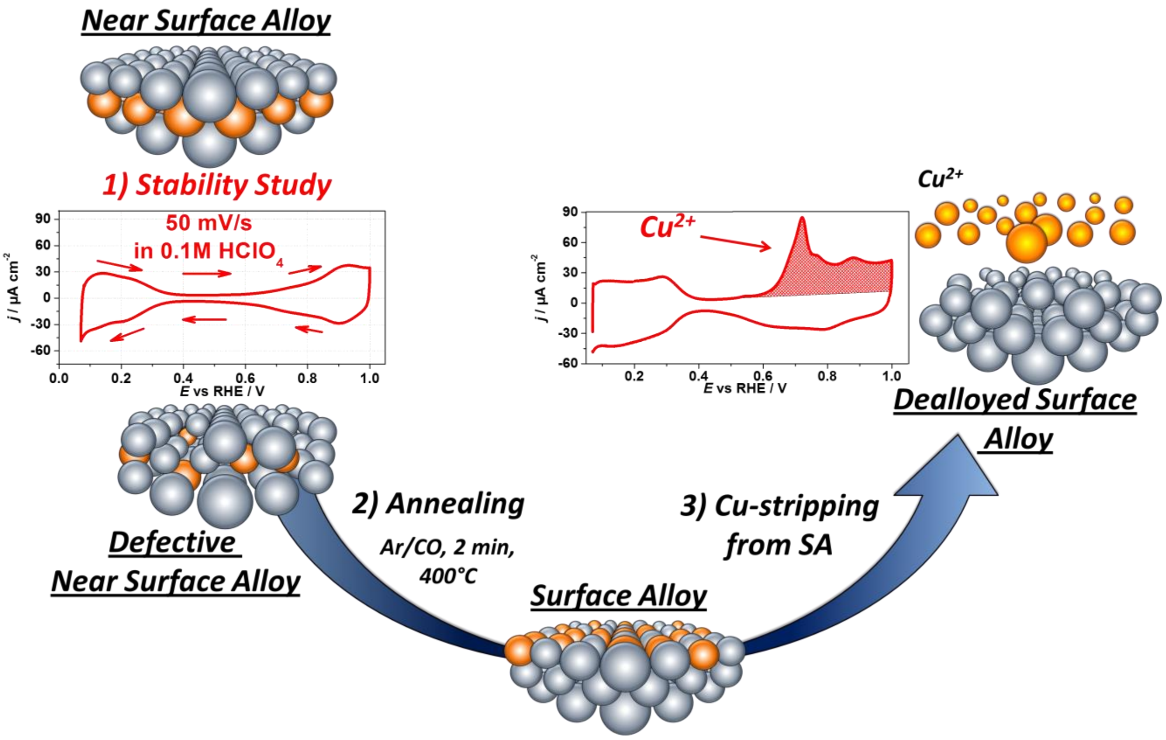


**Supplementary Figure 9.** The overall strategy employed to explore the effect of potential cycles on the stability of Cu-Pt(111) NSAs. 1) Stability study: potential cycles in the range from 0.05 V – 1.00 V vs. RHE; scan rate 50 mV $s^{-1}$ in Ar-saturated 0.1 M $HClO_4$, 2) Defective NSA is converted into Cu-Pt(111) SA by annealing it in a stream of CO at 400°C for 2 min, 3) Cu oxidation from Cu-Pt(111) SA in Ar-saturated 0.1 M $HClO_4$, scan rate d$E$/d$t$ = 50 mV $s^{-1}$.

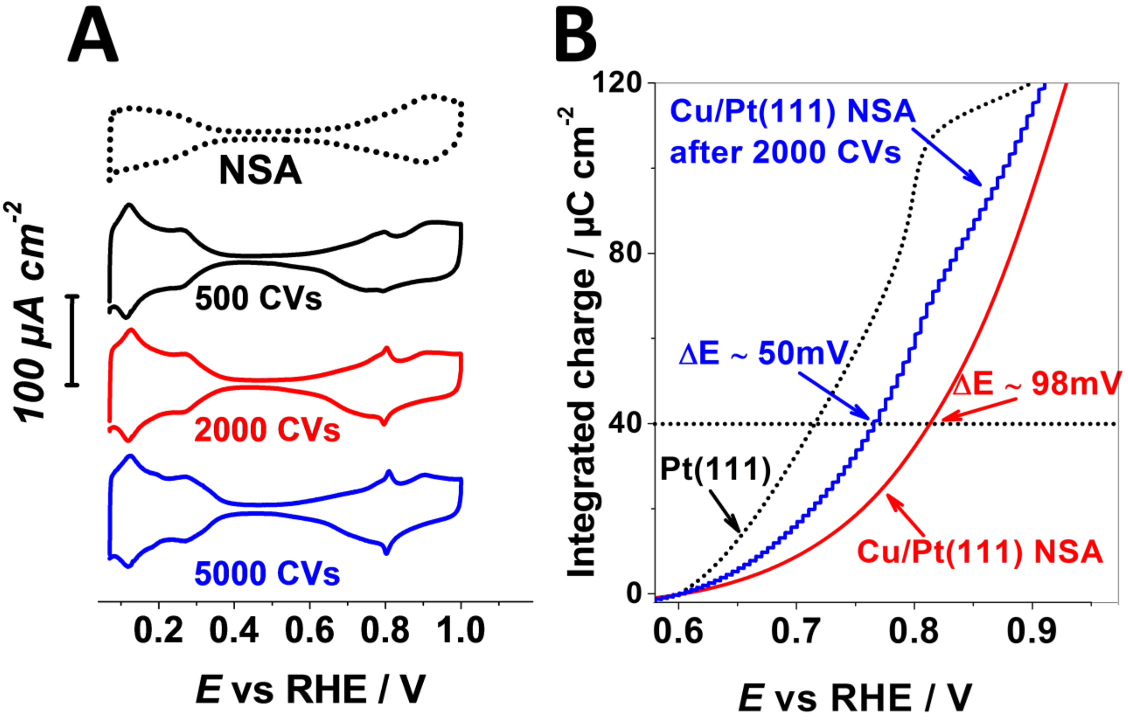


**Supplementary Figure 10**. Stability tests. A) A series of CVs for testing the stability of a freshly-prepared NSA (1 cycle, dotted line) in Ar-saturated 0.1 M $HClO_4$ after 500 (black solid line), 2000 (red) and 5000 cycles (blue). Scan rate: 50 mV $s^{-1}$, potential range from 0.05 V to 1.0 V vs RHE. B) Integrated anodic part of the CVs for Pt(111) (dotted line), Cu-Pt(111) NSA before cycling (red) and Cu/Pt(111) NSA after 2000 cycles (blue) in the potential range from 0.05 V to 1.0 V vs RHE.

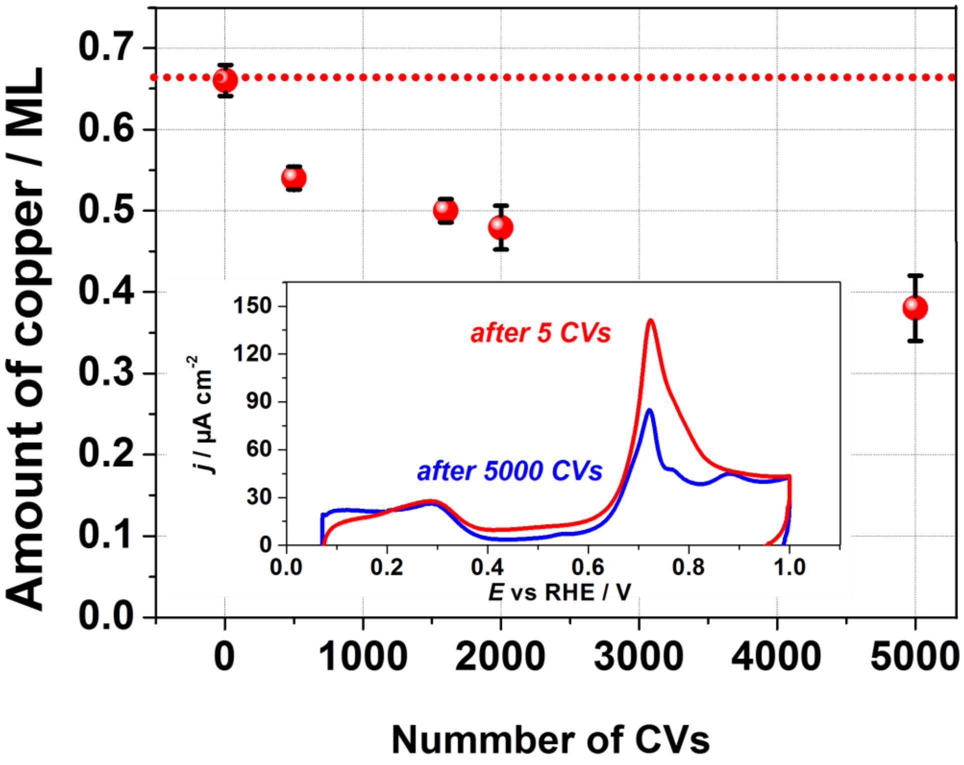


**Supplementary Figure 11.** Stability tests. Correlation between the amount of Cu remaining after stability tests and the numbers of cycles; the electrode was cycled in the potential range between 0.05 V and 1.0 V vs. RHE. The inset shows the anodic parts of the CVs demonstrating Cu stripping from Cu-Pt(111) SAs after cycling Cu-Pt(111) near-surface alloy (5 cycles – red, 5000 cycles – blue) in Ar-saturated 0.1 M $HClO_4$, d*E*/d*t* = 50 mV $s^{-1}$.

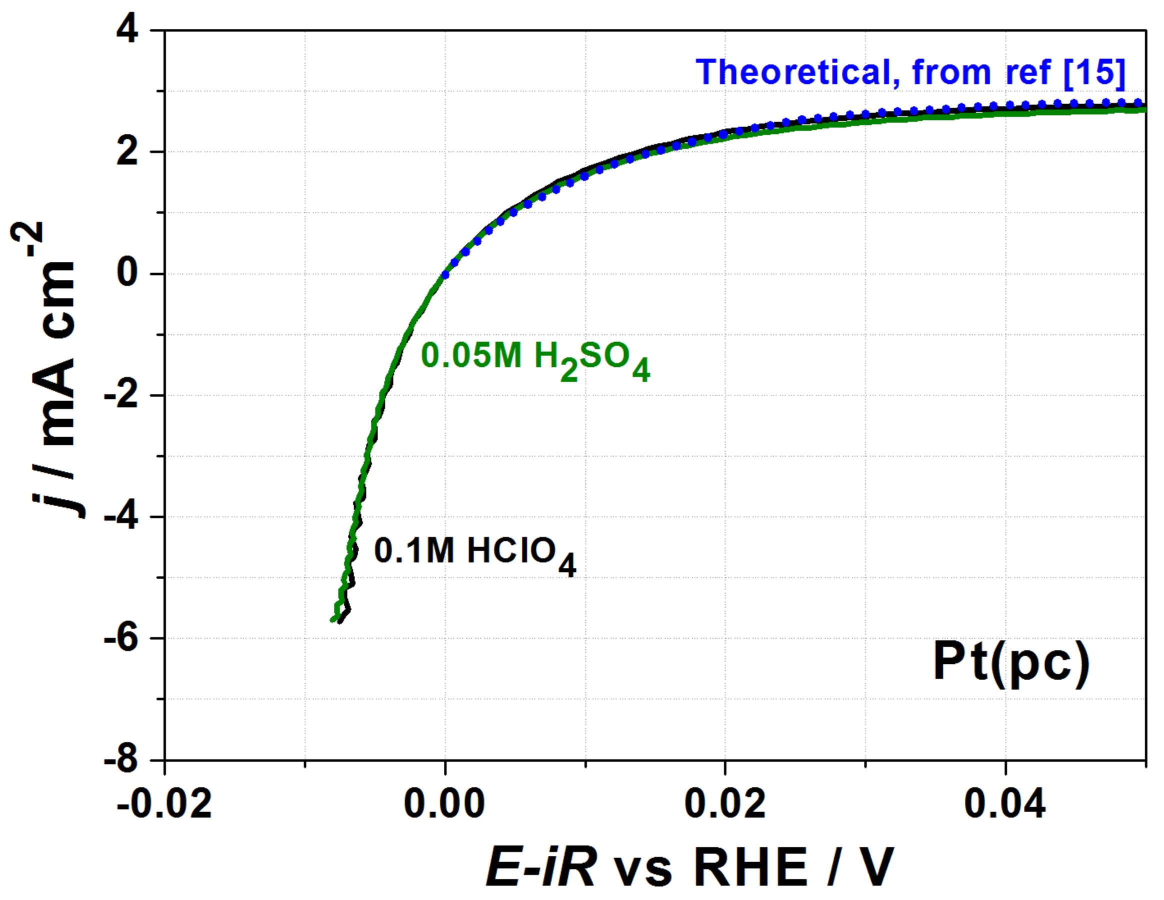


**Supplementary Figure 12.** Activities of Pt(pc) in perchloric and sulfuric acid electrolytes after the iR-correction.

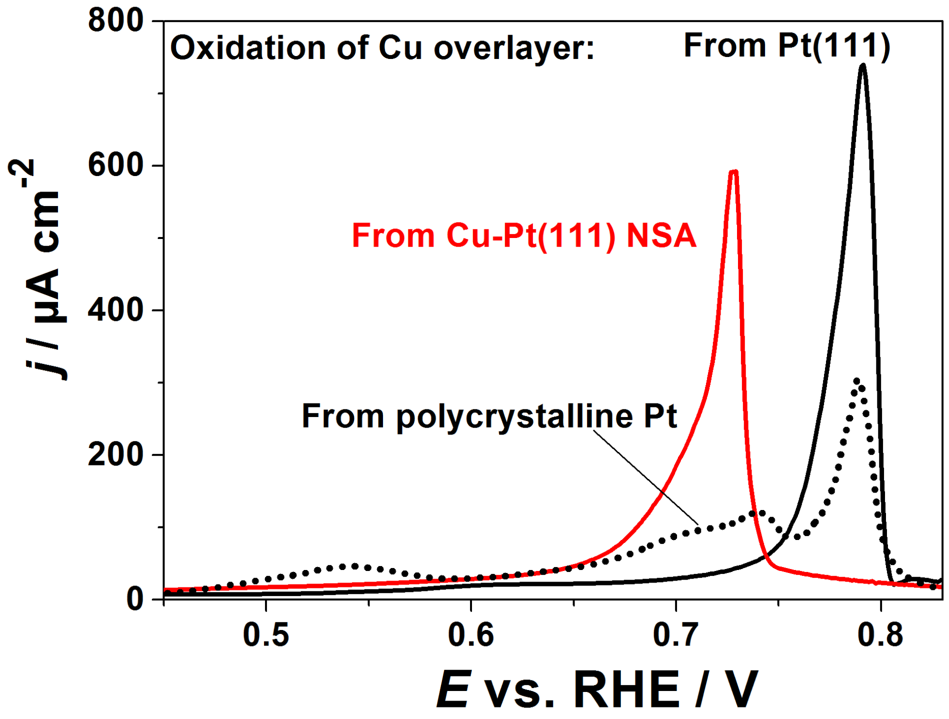


**Supplementary Figure 13.** Cu monolayer oxidation from different surfaces. Anodic parts of voltammograms are shown (d$E$/d$t$ = 50 mV $s^{-1}$) characterizing oxidation of a Cu monolayer from Pt(111), polycrystalline Pt and Cu-Pt(111) NSA (the most active surface towards HER).

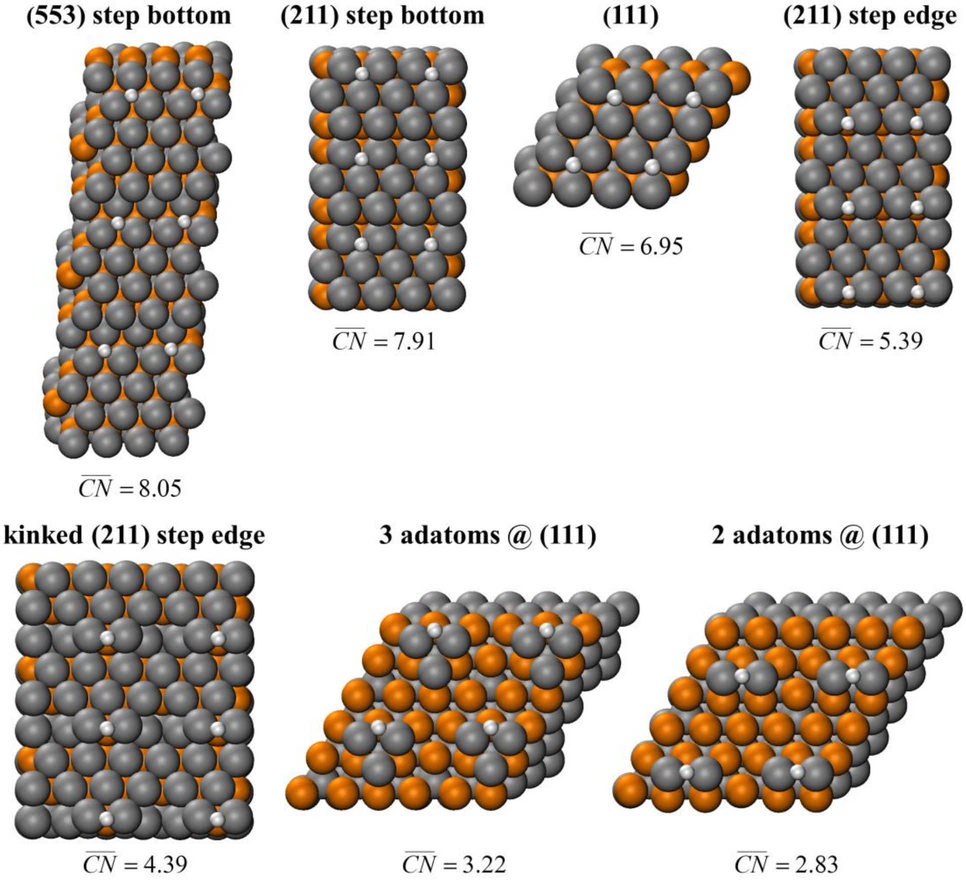


**Supplementary Figure 14.** Top views of the most stable adsorption configurations of *H (white color) on the various adsorption sites considered in this study for the Cu-Pt NSAs. In all cases, the second layer contains 1ML Cu. The adsorption configurations on Pt surfaces are similar.

## Supplementary Tables

**Supplementary Table 1.** List of k-point samplings used in this study and the generalized coordination numbers of the surface sites where *H adsorbs.

| surface description | k-point sampling | $\overline{CN}_{H*}$ |
|---|---|---|
| (√3×√3 R30º) 111 | 12×12×1 | (9×9 + 6×12)/22 = 6.95 (fcc) |
| 2-atom-wide 211 | 10×8×1 | (4×7 + 2×9 + 2×10 + 9×12)/22 = 7.91 (bottom) |
| 2-atom-wide 211 | 10×8×1 | (2×7 + 3×9 + 2×10 + 3×12)/18 = 5.39 (edge) |
| 3-atom-wide 211 kinked | 7×8×1 | (2×8 + 1×9 + 2×10 + 2×11 + 1×12)/18 = 4.39 (edge) |
| 2-atom-wide 553 | 10×5×1 | (2×7 + 5×9 + 2×11 + 8×12)/22 = 8.05 (bottom) |
| 3 adatoms @ (3×3) 111 hcp | 7×7×1 | (6×10 + 1×12)/22 = 3.27 (hollow) |
| 3 adatoms @ (3×3) 111 fcc | 7×7×1 | (1×5 + 2×10 + 3×11)/18 = 3.22 (bridge) |
| 2 adatoms @ (3×3) 111 | 7×7×1 | (4×10 + 1×11)/18 = 2.83 (bridge) |

## Supplementary Methods

We used the setup shown in Supplementary Figure 1 and also described in [1]. The cell has a reference electrode (RE), a counter electrode (CE) (platinum wire, 10 cm, ø 1 mm), a platinum(111) single crystal working electrode (WE) using a hanging meniscus configuration and a ‘‘dummy’’ platinum electrode. The temperature of the WE during thermal treatment is controlled by a K-type (Ni/Cr, Ni/Al) thermocouple integrated into a specially designed shaft which also enables rotating disk electrode (RDE) experiments. To avoid contamination of the electrolyte, the RE is placed in a separate compartment connected through a so-called Luggin capillary. To allow fast saturation of the working electrolyte with different gases the electrochemical cell has an inlet separated by a protecting glass wall. This reduces disturbing effects of bubble formation. The electrolyte volume in the cell can be varied between 10 and 50 ml. The electrolyte is delivered from a separate compartment using a glass connection. The preconditioning container is equipped with independent gas inlets and outlets for saturation of the electrolyte with different gases. Before the experiments, the cell, the preconditioning container and other glass parts were cleaned with Piranha solution (3:1 mixture of $H_2SO_4$ and 30% $H_2O_2$) for 12 h. The solution was then drained and all glass parts carefully rinsed with copious amounts of ultra-pure water. Next, the cell was cleaned with 50% HF/$H_2O$ solution, followed by multiple rinsing with ultra-pure water. The Luggin capillary was cleaned by multiple boiling/rinsing procedures. The CE was prevented from contact with piranha solution and was cleaned by multiple flame annealing and subsequent rinsing with ultra-pure water.

The procedure for preparation of Pt(111) single-crystal electrodes consists of two steps: (i) electrochemical cleaning of the electrode and (ii) an annealing/cooling step in reductive atmosphere. Electrochemical cleaning has been performed in 0.1 M $HClO_4$ by multiple cycling of the electrode in the potential region from 0.05 V to 1.0 V vs. RHE for ca. 30 min. In the next step, the electrode was rinsed with ultra-pure water and dried in a stream of Ar at room temperature. To restore the Pt(111) facets at the crystal surface the electrode was repeatedly annealed using the inductive heater and then cooled down in a flow of Ar/CO (0.1%). The same procedure was used to prepare the polycrystalline Pt electrode.

Supplementary Figure 2 illustrates a typical cyclic voltammogram obtained for a Pt(111) single crystal and a polycrystalline Pt electrode in 0.1 M $HClO_4$ prepared using the facilities of the setup. The presence of characteristic features which are extremely sensitive to

the presence of defects and cleanliness of the system, for instance sharp peaks at around 0.8 V and 1.07 vs RHE and well-defined H-UPD region shown in Supplementary Figure 2, has been used to evaluate the quality of the platinum(111) surface.

Modifications in the very topmost atomic layer are of paramount importance for the catalytic performance as this is where the reactants adsorb. The strategy used to modify the surface binding properties of platinum(111) electrodes is schematically shown in Supplementary Figure 3 and was discussed in detail elsewhere [1-8]. To briefly summarize: in the first step, a Cu pseudomorphic overlayer was deposited on a Pt(111) or Pt(pc) electrode. Deposition of a Cu pseudomorphic overlayer on Pt electrodes was performed in 0.1 M $HClO_4$ containing 4 mM $Cu^{2+}$ ions. The freshly-prepared Pt electrode was introduced into the electrolyte at a potential of 0.33 V vs RHE and it was cycled in the potential range from 0.32 V to 1.0 V vs. RHE (scan rate: 50 mV $s^{-1}$). To deposit the Cu pseudomorphic overlayer the potential was held at 0.33 V vs. RHE. After at least 3 min the dummy electrode was connected and the electrolyte was replaced by ultra-pure water.

After multiple rinsing of the cell and the modified electrode with ultra-pure water (under uninterrupted potential control), the working electrolyte (0.1 M $HClO_4$) was introduced into the electrochemical cell and the Cu pseudomorphic overlayer on Pt(POL@Pt) was characterized (Supplementary Figure 4). Subsequently, the POL@Pt was rinsed with ultra-pure water while in potential control using the "dummy" electrode at 0.33 V vs. RHE and dried in a $H_2$ (5.0) stream. Next, the working electrode was disconnected from the potentiostat and annealed at 400°C for 2 min in $H_2$ atmosphere using the inductive heater. After cooling down the WE, Ar sat. 0.1 M $HClO_4$ was introduced into the cell and the resulting near-surface alloy (NSA) was characterized (Supplementary Figure 4). Then, the NSA was transformed into a surface alloy (SA). For that, the NSA was rinsed with ultra-pure water, dried in a stream of Ar/CO (0.1%) and annealed at 400°C for 2 min using the inductive heater. After cooling down in an Ar stream the electrode was introduced into the working electrolyte under potential control (0.05 V vs. RHE) using the dummy electrode and characterized, see Supplementary Figure 4. The characterization of the NSA and SA surfaces using scanning probe techniques reveals only slight strains in the topmost layer [13]. However, unambiguous distinction between Cu and Pt using STM or AFM is difficult as these techniques are not sufficiently selective. Therefore, only AR-XPS (see [2]) and electrochemical means enforced by thermal annealing have been successfully used to confirm the relative position of Cu. As reported recently [1-7,10] cyclic voltammetry performed in 0.1

M $HClO_4$ allows unambiguous "fingerprint" distinction between Pt(111), Cu-Pt(111) NSA, Cu-Pt(111) SA, and Cu overlayers. Therefore, voltammetry was used to ensure that the required surface was obtained.

Another approach to tune interface binding properties is to vary the concentration of the solute element in the subsurface region. To achieve different concentrations of Cu in the second atomic layer, the amount of Cu deposited on the Pt electrode was adjusted by varying the deposition time between 30 s and 10 min for 0.2 ML and 1.0 ML, respectively. The Cu deposition was performed in 0.1 M $HClO_4$ containing 2 mM $Cu^{2+}$. To deposit X ML ($0<X<1$) of Cu the potential was held at 0.58 V vs. RHE. After a desired amount of Cu was deposited, the dummy electrode was connected and the electrolyte was replaced by ultra-pure water. In the next step, the X ML Cu-modified electrode was placed in the heating compartment and annealed at 400°C for 2 min in $H_2$ (5.0) atmosphere as shown schematically in Supplementary Figure 5A resulting in NSAs with different Cu concentration in the second atomic layer. Supplementary Figure 5B shows the oxidation peaks for Pt(111) after deposition of 0.2, 0.4, 0.6, 0.8 and 1.0 ML of Cu in Ar-saturated 0.1 M $HClO_4$.

Incorporation of Cu atoms into the subsurface of Pt affects the surface binding properties massively resulting in a surface that binds *H and *OH weaker than unmodified Pt(111) (Supplementary Figure 6A). In addition, changes in the binding energies correlate with the subsurface concentration of Cu. The changes in the *H and *OH binding are good examples, and can be used as evidence for different subsurface Cu concentrations [9,10]. Note that incorporation of 1.0 ML of Cu into Pt(111) weakens the Pt-H interaction leading to ~50% lower H coverage (Supplementary Figure 6B,C).

Another evidence for changes in the Cu content in the Pt subsurface region are the changes in the adsorption of (bi)sulfate ions on NSA in 0.05 M $H_2SO_4$. Supplementary Figure 7A shows theoretical trends in adsorption enthalpies of $*SO_4$ and *OH as a function of subsurface Cu content [10]. Although in both cases the increase in Cu content decreases the adsorption enthalpies, the effect is qualitatively and quantitatively different for both species: while the trend is roughly linear with a modest slope for *OH, the effect for $*SO_4$ is even more pronounced with an approximately parabolic trend. According to Supplementary Figure 7A, for subsurface Cu content below ~0.5 ML, $*SO_4$ and *OH adsorption enthalpies are similar, which suggests competitive adsorption when sulfate ions are in solution [10]. However, the adsorption of $*SO_4$ is dramatically suppressed as compared with that of *OH species when the Cu content increases above 0.5 ML favoring adsorption of the latter. This is

consistent with the experimental results in Supplementary Figure 7B where the black arrow indicates that *OH adsorption occurs when the Cu content in the second atomic layer exceeds 0.5 ML.

The formation of palladium pseudomorphic overlayers on both Pt(111) and polycrystalline Pt electrodes was performed in 0.1 M $HClO_4$ containing 0.4 mM $PdCl_4^{2+}$. The freshly-prepared electrodes (Pt(111) and Pt(pc)) were introduced into the electrolyte at a potential of 0.6 V vs RHE and the potential was held for 10 min. Afterwards, the Pd-modified electrode was rinsed with ultra-pure water and characterized by means of cyclic voltammetry as shown in Supplementary Figure 8. The results are consistent with those reported by Feliu et al. in ref [11].

Supplementary Figure 9 shows the overall strategy employed to explore the stability of Cu-Pt(111) near-surface alloys in Ar-saturated 0.1 M $HClO_4$ at room temperature. Stability tests were performed by cycling the electrode in the potential range of 0.05 V to 1.0 V vs. RHE (number of cycles: 5, 500, 1600, 2000 and 5000) [12].

The degradation of the NSA was monitored using cyclic voltammetry (Supplementary Figure 10). Previous studies showed that on Cu/Pt(111) NSA ~2/3 ML Cu is stabilized in the subsurface [2,13]. This feature is used to evaluate its stability. By cycling the NSA above +0.34 V vs. RHE any Cu atom exposed to the electrolyte is oxidized. The Pt outermost layer on the NSA prevents Cu oxidation when cycling even up to higher values. To quantify the amount of Cu left after stability tests, the NSA was converted into a SA using the procedure above. Afterwards, the amount of Cu in the SA was determined by integrating the Cu oxidation peak compared to the value of ~2/3 ML Cu. Catalyst degradation leads to Cu dissolution, monitored using cyclic voltammetry (Supplementary Figure 10A). The loss of Cu affects the binding of the catalyst towards *H and *OH (Supplementary Figure 10A). The effect of dealloying/degradation of Cu-Pt(111) on *OH binding appears in Supplementary Figure 10B.

Supplementary Figure 11 shows the anodic parts of the CVs for Cu stripping from the SA in Ar-saturated 0.1 M $HClO_4$ after 5 and 5000 cycles. Using the charge obtained for Cu oxidation and the theoretical value required for the deposition/stripping of a fully discharged monolayer of Cu, namely 440 μC $cm^{-2}$ [14], we determine the remaining amount of Cu. After 5 cycles, a charge of ~300 μC $cm^{-2}$ was obtained, which corresponds to ~0.68 ML Cu and is close to the value of ~2/3 ML expected for the fresh NSA. In contrast, for the sample cycled

5000 times, a charge of ~176 μC $cm^{-2}$ was determined which is equal to ~0.4 ML of Cu. This shows that cycling the NSA in the potential range from 0.05 V to 1.0 V vs RHE leads to partial surface corrosion and subsequent dissolution of Cu atoms from the subsurface. In addition, the correlation between the amount of Cu determined for the SA and the number of cycles applied to the NSA is shown. After 5000 cycles, ~40% of Cu incorporated into the second layer of NSA is lost due to the defects introduced to the Pt outermost layer.

Hydrogen evolution in acidic media is one of the fastest electrocatalytic reactions known to date. However, there are certain discrepancies in the literature regarding the activity of Pt(111) and Pt(pc). Normally, Pt(111) demonstrates significantly lower activities towards HER/HOR as compared to Pt(pc) in acidic media. Recently, Sheng et al [15] performed benchmarking of the HER/HOR activities of Pt(pc), particularly in acidic media. HOR appeared to be diffusion-controlled. Quantification of the HER activity after iR-correction appeared to be non-trivial. We recently explored some experimental aspects of quantification of the HER activities [16]. Even small errors during electrochemical impedance spectroscopy lead to unacceptable errors in the quantification of HER activities. Therefore, we rather compare the materials under identical conditions. Nevertheless, Supplementary Figure 12 demonstrates that our experimental protocol is capable of reaching the benchmarks reported in the literature and additionally confirms that the HER/HOR activities should be the same in acidic sulfuric and perchloric aqueous electrolytes.

Adsorption and subsequent oxidation of Cu monolayers often reveal different affinity of different surface sites towards many adsorbates: these experiments are roughly comparable to the temperature-programmed techniques used in surface science. Cu can form pseudomorphic overlayers at the surface of Pt electrodes. This is very convenient as compared to the adsorption of e.g. CO where the fractional coverage of CO also depends on the electronic structure of the surface. Different relative positions of Cu stripping peaks give the information about energetic/adsorption properties of different centers. Trends in adsorption energies for the same type of sites for different surfaces are approximately similar [17]. Supplementary Figure 13 shows that the surface of Pt(111) binds Cu adatoms stronger than Cu-Pt(111) NSAs, similar to the situation with *H (see Figure 2 in the manuscript). However, in the case of *H the maximal fractional coverage on Cu-Pt(111) is 50% smaller as compared to Pt(111). In contrast Cu atoms "explore" all Pt atoms at the surface.

Importantly, Cu stripping experiments performed using polycrystalline Pt (Supplementary Figure 13) demonstrate that due to coordination issues (see explanations

below regarding $\overline{CN}$ and the main text) the surface of polycrystalline Pt has sites with adsorption properties similar to those of Pt(111) and Cu-Pt(111) NSAs. This further explains why polycrystalline Pt is more active than Pt(111) but less active than the NSAs.

We performed DFT calculations using the Vienna Ab Initio Simulation Package [18], with the projector augmented-wave (PAW) method [19] and the PBE exchange-correlation functional [20]. The extended surfaces contained four metal layers, the two topmost of which as well as the adsorbates were free to relax in all directions, whereas the 2 bottommost layers were fixed at the optimized bulk interatomic distances, which are 2.81 Å within the PBE formalism for Pt. The atomic relaxations were performed using the conjugate-gradient scheme with a plane-wave cut-off of 450 eV, until the maximum force on any free atom was below 0.01 eV Å$^{-1}$. Since the differences in *H adsorption energies between various adsorption sites are small, we used dense k-point meshes, see Supplementary Table 1.

The vacuum layer between images in extended surfaces was larger than 14 Å and dipole corrections were applied. An electronic temperature of 0.2 eV was used, and the energies were extrapolated to T = 0 K. $H_2$ was calculated in a cubic box of 15 Å × 15 Å × 15 Å using a gamma point distribution and an electronic temperature of 0.001 eV. The computational hydrogen approach [21] allows for the use of the energetics of gas-phase hydrogen instead of those of protons and electrons in an electrochemical environment, as they are related by the following equilibrium:

$$\tfrac{1}{2}H_2 \leftrightarrow H^+ + e^- \qquad \text{(S1)}$$

Thus, if the adsorption of protons is described in the following way:

$$* + H^+ + e^- \rightarrow *H \qquad \text{(S2)}$$

Then their adsorption energies are calculated as follows:

$$\Delta G_H = G_{*H} - G_* - \tfrac{1}{2}G_{H_2} \qquad \text{(S3)}$$

In these equations * is a free adsorption site. The free energies of the adsorbates are approximated as:

$$G = E_{DFT} + ZPE - TS \qquad \text{(S4)}$$

$E_{DFT}$ is the DFT-calculated total energy. *ZPE* is the zero-point-energy correction obtained through vibrational-frequency analysis, using the harmonic oscillator approximation. The ZPE of *H on Pt is 0.15 eV, while it is 0.27 eV for $H_2$. The entropy correction for *H is 0.01 eV at 300 K, while it is 0.40 eV for $H_2$. Supplementary Figure 14 shows the adsorption

sites of *H on the various facets considered in this study and their corresponding generalized coordination numbers, also given in Supplementary Table 1.

The microkinetic model used to estimate the HER current densities of the sites under study was taken from Norskov et al [22]. In it, the current densities for $\Delta G_H > 0$ are given by the following formula:

$$i_0 = \frac{-ec \cdot k_0 \cdot e^{-\Delta G_H / k_B T}}{1 + e^{-\Delta G_H / k_B T}} \quad \text{(S5)}$$

The current densities for $\Delta G_H < 0$ are given as:

$$i_0 = \frac{-ec \cdot k_0}{1 + e^{-\Delta G_H / k_B T}} \quad \text{(S6)}$$

In these equations $-ec = 1.602 \times 10^{-19} C$ is the electron charge, $T = 300\ K$, the Boltzmann constant is $k_B = 8.617 \times 10^{-5}$ eV $K^{-1}$, and $k_0 = 2 \times 10^{17} s^{-1} cm^{-2}$.

## Supplementary References


[1] Tymoczko J., Schuhmann W., Bandarenka A.S. A versatile electrochemical cell for the preparation and characterisation of model electrocatalytic systems. *Phys. Chem. Chem. Phys.* **15**, 12998-13004 (2013).

[2] Bandarenka A.S., Varela A.S., Karamad M., Calle-Vallejo F., Bech L., Perez-Alonso F.J., Rossmeisl J., Stephens I.E.L., Chorkendorff I. The design of an active site towards optimal electrocatalysis: Overlayers, surface alloys and near-surface alloys of Cu/Pt(111). *Angew. Chem. Int. Ed*. **51**, 11845-11848 (2012).

[3] Bondarenko A.S., Stephens I.E.L., Chorkendorff I. A cell for the controllable thermal treatment and electrochemical characterisation of single crystal alloy electrodes. *Electrochem. Commun*. **23**, 33-36, (2012).

[4] Bondarenko A.S., Stephens I.E.L., Bech L., Chorkendorff I. Probing Adsorption Phenomena on a Single Crystal Pt-alloy Surface under Oxygen Reduction Reaction Conditions. *Electrochim. Acta* **82**, 517-523 (2012).

[5] Stephens I.E.L., Bondarenko A.S., Perez-Alonso F.J., Calle-Vallejo F., Bech L., Johansson T.P., Jepsen A.K., Frydendal R., Knudsen B.P., Rossmeisl J., Chorkendorff I. Tuning the activity of Pt(111) for oxygen electroreduction by subsurface alloying. *J. Am. Chem. Soc*. **133**, 5485-5491, (2011).

[6] Tymoczko J., Schuhmann W., Bandarenka A.S. Position of Cu atoms at the Pt(111) electrode surfaces controls electrosorption of (bi)sulfate anions from $H_2SO_4$ electrolytes. *ChemElectroChem* **1**, 213-219, (2014).

[7] Maljusch A., Henry J.B., Tymoczko J., Bandarenka A.S., Schuhmann W. Characterisation of non-uniform functional surfaces: towards linking basic surface properties with electrocatalytic activity. *RSC Adv*. **4**, 1532-1537 (2014).

[8] Henry J.B., Maljusch A., Huang M., Schuhmann W., Bondarenko A.S. Thin-film Cu-Pt(111) near-surface alloys: active electrocatalysts for the oxygen reduction reaction. *ACS Catal*. **2** 1457-1460 (2012).

[9] Bandarenka A.S., Koper M.T.M., Structural and electronic effects in heterogeneous electrocatalysis: towards a rational design of electrocatalysts. *J. Catal.* **306**, 11-24 (2013).

[10] Tymoczko J., Calle-Vallejo F., Colic V., Koper M.T.M., Schuhmann W., Bandarenka A.S., Oxygen Reduction at a Cu Modified Pt(111) Model Electrocatalyst in Contact with NAFION polymer. *ACS Catal*. **4**, 3772-3778 (2014).

[11] Álvarez B., Climent V., Rodesa A., Feliu J.M., Potential of zero total charge of palladium modified Pt(111) electrodes in perchloric acid solutions. *Phys. Chem. Chem. Phys*. **3**, 3269-3276 (2001).

[12] Tymoczko J., Calle-Vallejo F., Colic V., Schuhmann W., Bandarenka A.S. Evaluation of the electrochemical stability of model Cu-Pt(111) near-surface alloy catalysts. *Electrochim. Acta* **179**, 469-474 (2015)

[13] Knudsen J., Nilekar A.U., Vang R.T., Schnadt J., Kunkes E.L., Dumesic J.A., Mavrikakis M., Besenbacher F. A Cu/Pt near-surface alloy for water-gas shift catalysis. *J. Am. Chem. Soc*. **129**, 6485-6490 (2007).

[14] Herrero E., Buller L.J., Abruña H.D. Underpotential deposition at single crystal surfaces of Au, Pt, Ag and other materials, *Chem. Rev*. **101**, 1897-1930 (2001).

[15] Sheng W.C., Gasteiger H.A., Shao-Horn Y. Hydrogen oxidation and evolution reaction kinetics on platinum: acid vs alkaline electrolytes. *J. Electrochem. Soc.* **157**, B1529-B1536 (2010).

[16] Čolić V., Tymoczko J., Maljusch A., Ganassin A., Schuhmann W., Bandarenka A.S. Experimental aspects in benchmarking of the electrocatalytic activity. *ChemElectroChem*, **2**, 143-149 (2015).

[17] Calle-Vallejo F., Loffreda D., Koper M.T.M., Sautet P. Introducing structural sensitivity into adsorption–energy scaling relations by means of coordination numbers, *Nature Chem*. **7**, 403-410 (2015).

[18] Kresse G., Furthmüller J. Efficient iterative schemes for ab initio total-energy calculations using a plane-wave basis set, *Phys. Rev. B,* **54**, 11169-11186 (1996).

[19] Kresse G., Joubert D., From ultrasoft pseudopotentials to the projector augmented-wave method. *Phys. Rev. B,* **59**, 1758-1775 (1999).

[20] Perdew J.P., Burke K., Ernzerhof M. Generalized gradient approximation made simple. *Phys. Rev. Lett.* **78**, 1396 (1997).

[21] Nørskov J.K., Rossmeisl J., Logadottir A., Lindqvist L. Origin of the overpotential for oxygen reduction at a fuel-cell cathode, *J. Phys. Chem. B*, **108**, 17886-17892 (2004).

[22] Nørskov J.K., Bligaard T., Logadottir A., Kitchin J.R., Chen J.G., Pandelov S., Stimming U. Trends in the exchange current for hydrogen evolution. *J. Electrochem. Soc*. **152** (3), J23-J26 (2005)
